# Behavior of Ternary Mixtures of Hydrogen Bond Acceptors and Donors in Terms of Band Gap Energies

**DOI:** 10.3390/ma14123418

**Published:** 2021-06-20

**Authors:** Alberto Mannu, Francesca Cardano, Salvatore Baldino, Andrea Fin

**Affiliations:** 1 Department of Chemistry, University of Turin, Via Pietro Giuria 7, I-10125 Turin, Italy; francescacardano@gmail.com (F.C.); salvatore.baldino@unito.it (S.B.); 2 Department of Drug Science and Technology, University of Turin, Via Pietro Giuria 9, I-10125 Turin, Italy; andrea.fin@unito.it

**Keywords:** eutectic mixtures, hydrogen bond acceptor, hydrogen bond donor, design of experiments, Tauc plot, band gap energy

## Abstract

Three ternary mixtures composed by choline chloride (ChCl), ethylene glycol (EG), and a second hydrogen bond donor (HBD) as ethanol (A), 2-propanol (B), and glycerol (C) were studied in terms of composition related to the band gap energy (BGE). A Design of Experiments (DoE) approach, and in particular a *Simple Lattice* three-components design, was employed for determining the variation of the BGE upon the composition of each system. UV-VIS analysis and subsequent Tauc plot methodology provided the data requested from the DoE, and multivariate statistical analysis revealed a drop of the BGE in correspondence to specific binary compositions for systems A and B. In particular, a BGE of 3.85 eV was registered for the mixtures ChCl/EtOH (1:1) and ChCl/2-propanol (1:1), which represents one of the lowest values ever observed for these systems.

## 1. Introduction

Hydrogen bond-based systems have been affirmed during the last 20 years as one of the most recurrent topics in the scientific literature [1]. In particular, the possibility to combine in a eutectic molar ratio Hydrogen Bond Acceptors (HBAs) and Hydrogen Bond Donors (HBDs) and form liquid mixtures at room temperature with increased solvent ability, which was reported for the first time by Abbott et al. in 2003 (choline chloride/urea 1:2) [2], created opportunity for many applications in several research and industrial sectors. Since the first paper of Abbot about the topic, dozens of such systems have been developed, characterized, and studied in terms of physical–chemical properties [3,4,5]. These systems are of particular interest when the molar ratio between HBAs and HBDs produces a drop of the melting point which produces experimental results deeper than the expected theoretical ones [3,6]. When this specific combination takes place, a concomitant increased solvent ability is also observed [7], and for this reason, the acronym DESs has been proposed, which stands for Deep Eutectic Solvents. Through the years, many researchers have been trigged by the possibility of engineering DESs by choosing opportune combinations of HBAs and HBDs and by finding the best molar ratio between them [6]. At the molecular level, most of the DESs can be described according to the hole theory [8] and rationalized as systems made by an intense hydrogen bond network decorated with randomly distributed holes, where the ions can move along the network by jumping from one hole to another [9]. This supramolecular behavior gives to the system peculiar properties such as an increased density, a decreased viscosity, and a low conductivity [1]. On the basis of such characteristics, DESs have found many applications as media for biomass treatment [10], metal extraction [11], solvents for Volatile Organic Compounds (VOCs) [12,13], templates for ionothermal synthesis [14,15,16], or non-innocent solvents in organic synthesis [17,18,19,20,21], as well as additives in pharmaceutical formulations [1,22,23].

Recently, some preliminary studies have highlighted how the deep eutectic composition in mixtures of hydrogen bond acceptors and donors is related to a depression of the band gap energy [24,25] along with a drop of the structural disorder (Urbach energy) [26]. The possibility to tune such optical parameters results is of interest especially for the development of new liquid organic semiconductors.

In this context, the possibility to model the band gap energy (BGE) and monitoring its variation in ternary mixtures composed by one HBA (choline chloride, ChCl), a first HBD (ethylene glycol, EG), and a second HBD (ethanol-EtOH (A), 2-propanol (B), or glycerol-GLY (C)) is herein explored. In particular, a Design of Experiments (DoE) approach followed by multivariate analysis was employed to finally plot a surface representative of the variation of the BGE depending on the molar fraction ratio between the constituents of each system. According to the DoE, seven samples were prepared for each one of the three systems (A, B, C) for a total of 21 experiments, which were subjected to the graphical Tauc plot method for the determination of the BGE.

## 2. Experimental Section

### 2.1. General Synthetic Procedure

Chemicals were purchased from commercial sources and used as received. In particular, choline chloride (>98%) and 2-propanol (99.8%) were purchased from Merck KGaA, Darmstadt, Germany, ethanol (96%) and glycerol (99.6%) were purchased from VWR, and ethylene glycol (99%) was purchased from Carlo Erba. Finally, H_2_O was purified with a Millipore RiO_s_ 3 Water System.

The samples were prepared following this protocol: ChCl was weighted in a vial, and 10 wt % of water was added. Thus, one or two hydrogen bond donors were added, and the resulting mixture was stirred for 2 h at room temperature before the analysis.

### 2.2. Spectroscopic UV-VIS Analysis

The samples were analyzed in a pure form by UV-VIS spectrophotometry. The spectra were recorded in transmittance mode in a quartz cell (path length: 1.00 mm) with an Agilent Cary 60 UV-Vis Spectrophotometer.

### 2.3. Statistical Analysis

Design of Experiment (DoE): a *Simple Lattice* three-components design was settled up [27] Thus, for each ternary system, we prepared seven samples with the molar ratio as reported in Table 1.

Multivariate analysis, including the Analysis of the Variance (ANOVA) and the corresponding surface plots, was conducted with the Statgraphics Centurion v 18 software, Statgraphics Technologies, Inc. The Plains, Virginia.

## 3. Results and Discussion

Ternary systems A, B, and C were prepared by combining ChCl, EG, and a second HBD. The choice of the second HBD was driven by the affinity of alcohols with ChCl and EG; thus, EtOH, 2-propanol, and GLY were selected.

The aim of the research was to develop a tool for engineering a ternary mixture of HBAs and HBDs in terms of BGE. Thus, the first target was to model the variation of the BGE in each ternary system A, B, and C and to provide a suitable statistical instrument for describe these mixtures. In order to reach such goal, a DoE approach was used, and in particular, a *Simple Lattice* three-components mixture experiment was implemented [28].

Seven different molar combinations were prepared for each system, and the corresponding BGEs were determined by the known graphic UV-VIS-based Tauc plot method, following a procedure previous optimized by some of us [24].

In Table 1, the nomenclature corresponding to the systems prepared and subjected to UV-VIS analysis is reported.

**Figure 1:**
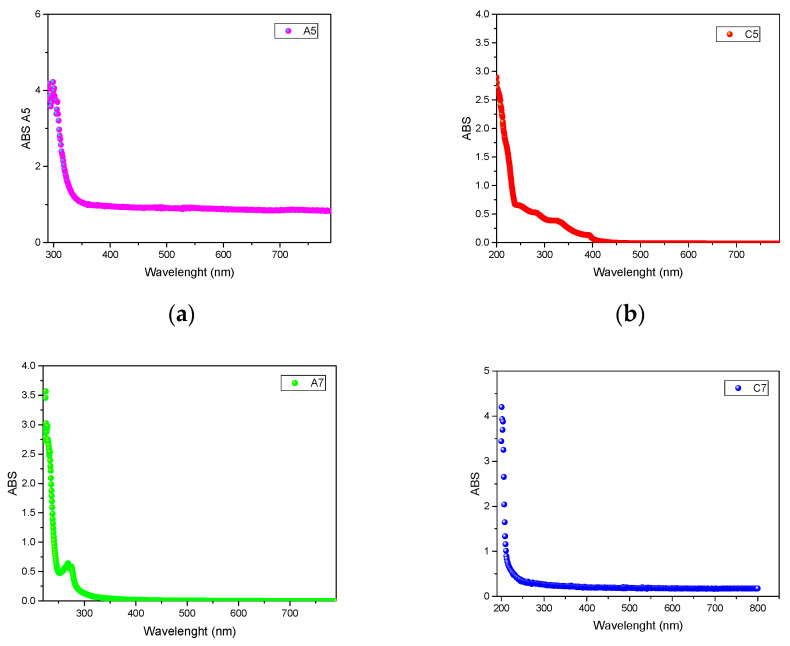
UV-VIS spectra of systems A5 (**a**), C5 (**b**), A7 (**c**), and C7 (**d**).

In Figure 1, the UV-VIS spectra of representative binary systems A5, C5, and the thernary ones A7, C7 are reported.

From a visual and qualitative analysis of the UV-VIS spectra of the systems reported in Figure 1, it is possible to notice some differences. In particular, systems A5 (a, ChCl/EG 1/1), and C7 (d, ChCl/EG/GLY 1/1/1) show the same UV-VIS behavior, suggesting that the addition of glycerol does not affect in a relevant way the optical characteristics of the system. On the other side, systems C5 (b, ChCl/GLY 1/1) and A7 (c, ChCl/EG/EtOH 1/1/1) reveal different absorbance spectra, with C5 showing multiple absorbance peaks between 200 and 400 nm, and A7 highlighting a relevant peak at 270 nm. Nevertheless, it is difficult to extrapolate trends and information from the analysis of the UV-VIS spectra. On the other side, the calculation of the band gap energy from the UV-VIS data can provide many information, which can be related with the structural effects of the constituents on the system.

Looking at the band gap data reported in Table 1, it is possible to highlight some trends. Each single component, measured in pure form, shows a relatively high BGE: BGE_ChCl_ 5.75 eV, BGE_EtOH_ 5.88 eV, BGE_GLY_ 5.23 eV, and BGE_EG_ 5.66 eV. As expected, when two HBDs are combined (1:1 molar ratio), no significant drop of the BGE is observed: BGE_EG/EtOH_ 6.05 eV (A6), BGE_EG/2-propanol_ 6.05 (B6), BGE_EG/GLY_ 5.17 eV (C6). In addition, no relevant differences were observed changing EtOH by 2-propanol (A6 vs. B6). As a matter of fact, the deepest reduction of the BGE was observed for the binary systems A5 and B5, which do not contain EG. This value of BGE, around 3.86 eV, falls in the range of interest for potential application as an organic liquid semiconductor. It is interesting to notice that the corresponding binary system C5, composed by ChCl and GLY, shows a BGA far away from the parent A5 and B5.

**Figure 2:**
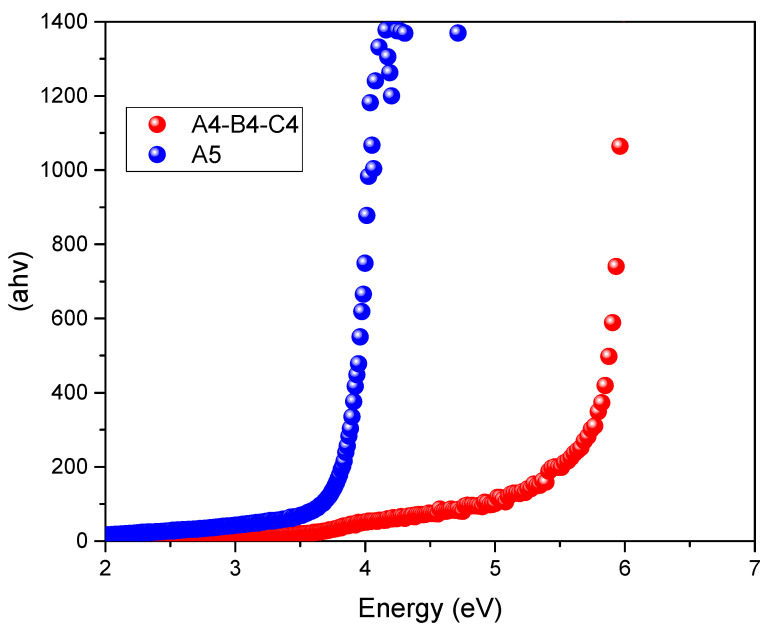
Tauc plots relative to the systems A4, B4, C4, ChCl/EG (1:1) and A5, ChCl/EtOH (1:1).

In Figure 2, the reduction of the BGE obtained by substituting EG with EtOH (A4, B4, and C4 vs. A5) is reported.

Each set of experiments (A, B, and C, Table 1) was processed according to the DoE procedure adopted to build a descriptive model of the variation of the BGE as a function of the molar ratio between the three constituents of the mixture.

### Multivariate Analysis of Systems A, B, and C

At first, system A composed by ChCl/EG/EtOH was analyzed with the aim to find the best statistical model that can represent the behavior of the mixture in terms of variation of the BGE. After a screening between linear, special cubic, and quadratic statistical models, the last one has been selected as the most accurate in describing the system. Full data details are reported in the Supporting Information file.

Starting from the selected quadratic model, the data obtained for the system A were subjected to the Analysis of the Variance (ANOVA), which gave the results reported in Table 2.

ANOVA analysis shows an R-squared value that indicates that the model as fitted explains 88.0759% of the variability in BGE. The adjusted R-squared statistic, which is more suitable for comparing models with different numbers of independent variables, is 28.4553%. The standard error of the estimate shows the standard deviation of the residuals to be 0.656277. The mean absolute error (MAE) of 0.195845 is the average value of the residuals. The Durbin–Watson (DW) statistic tests the residuals to determine if there is any significant correlation based on the order in which they occur in your data file. Since the *p*-value is greater than 5.0%, there is no indication of serial autocorrelation in the residuals at the 5.0% significance level.

Systems B and C were subjected to the same statistical treatment confirming the quadratic model as the best one.

For comparison purposes, the R-squared values of systems A, B, and C are reported in Table 3.

**Figure 3:**
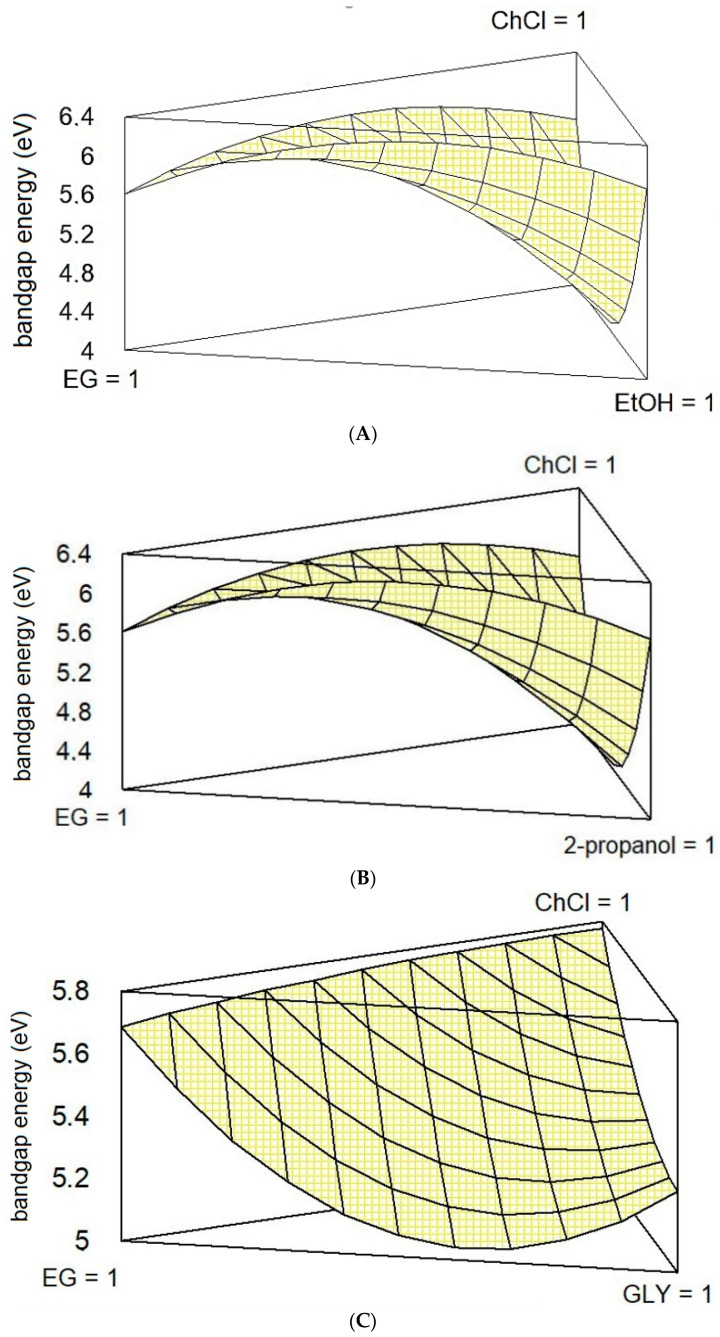
Estimated response surface for systems (**A**–**C**).

Once we determined the statistical parameters that better described the behavior of each system, it is possible to graphically represent them in the form of a responsive surface (Figure 3).

The surface responding plots reported in Figure 3 describe the variation of the systems A-C in terms of BGE. From a fist qualitative analysis, it is possible to notice that the shape of the surface that describes the behavior of the ternary mixture is very similar for systems A and B, while it changes for system C. This is mainly due to the previously commented different interaction between ChCl and GLY (C5) with respect to ChCl and EtOH (A5) or ChCl and 2-propanol (B5). This experimental behavior of C5, combined with lower maximum values of BGE for A6 and B6 (6.04 eV), determines a flatter surface. From the combined analysis of the plots reported in Figure 3, it is possible to conclude that only systems A and C show a consistent depression of the BGE, which indeed correspond to a binary system. No one of the three systems considered performs better (in terms of lower BGE) with a ternary composition. Thus, the increment of O-H bonds achieved with the introduction of a second HBD seems to negatively affect the eutectic nature of the mixture.

To the best of our knowledge, this study represents the first report about the variation of the BGE in ternary mixtures of HBAs and HBDs. In addition, the statistical model herein presented can be applied for optimizing other systems, even considering different parameters beyond the BGE.

## 4. Conclusions

Three ternary systems composed by ChCl, EG, and a second HBD (EtOH, 2-propanol, GLY) were studied in terms of variation of the BGE with respect to the molar ration of the former’s components. A statistical reliable model that describes the relationship between BGE and molar composition was built and described. The statistical multivariate analysis revealed, for the systems herein considered, that an excessive increasing of the O-H bonds affects the eutectic nature of the mixture, resulting in an increasing of the BGE. In addition, the combination between UV-VIS spectroscopy, Tauc plot method (for the band gap energy determination), and the Simple Lattice DoE followed by statistical multivariate analysis provide an easy and fast tool for engineering ternary mixtures of Hydrogen Bond Donors (HBDs) and Acceptors (HBAs). In fact, the combination of techniques reported allow mapping the variation of the band gap energy versus the molar composition of the ternary system. This procedure can be used for screening purposes with the target to select the best combination between HBDs and HBAs that provides the minimum band gap energy value.

## Figures and Tables

**Table 1:** BGEs and corresponding nomenclature for the systems A–C.

HBA (χ) ^1^	HBD (χ)	HBD (χ)	Name	BGE (eV)
ChCl (1)	EG (0)	EtOH (0)	A1	5.75
ChCl (0)	EG (1)	EtOH (0)	A2	5.66
ChCl (0)	EG (0)	EtOH (1)	A3	5.88
ChCl (0.5)	EG (0.5)	EtOH (0)	A4	5.86
ChCl (0.5)	EG (0)	EtOH (0.5)	A5	3.85
ChCl (0)	EG (0.5)	EtOH (0.5)	A6	6.05
ChCl (0.33)	EG (0.33)	EtOH (0.33)	A7	5.87
ChCl (1)	EG (0)	2-propanol (0)	B1	5.75
ChCl (0)	EG (1)	2-propanol (0)	B2	5.66
ChCl (0)	EG (0)	2-propanol (1)	B3	5.88
ChCl (0.5)	EG (0.5)	2-propanol (0)	B4	5.87
ChCl (0.5)	EG (0)	2-propanol (0.5)	B5	3.85
ChCl (0)	EG (0.5)	2-propanol (0.5)	B6	6.05
ChCl (0.33)	EG (0.33)	2-propanol (0.33)	B7	5.84
ChCl (1)	EG (0)	GLY (0)	C1	5.75
ChCl (0)	EG (1)	GLY (0)	C2	5.66
ChCl (0)	EG (0)	GLY (1)	C3	5.23
ChCl (0.5)	EG (0.5)	GLY (0)	C4	5.86
ChCl (0.5)	EG (0)	GLY (0.5)	C5	5.51
ChCl (0)	EG (0.5)	GLY (0.5)	C6	5.17
ChCl (0.33)	EG (0.33)	GLY (0.33)	C7	5.12

^1^ To each ChCl sample 10 wt % of water was added to make the samples measurable at UV-VIS.

**Table 2:** ANOVA for BGE of system A.

Source	Sum of Squares	Df	Mean Square	F-Ratio	*p*-Value
Quadratic model	3.1813	5	0.63626	1.48	0.5435
Total error	0.430699	1	0.430699		
Total (corr.)	3.612	6			

**Table 3:** R-squared for system A, B, and C.

System	Statistical Model	R-Squared (%)
A	quadratic	88.0759
B	quadratic	89.2872
C	quadratic	83.3460

## Data Availability

External data are not available.
